# Molecular Dissection of the Gene *OsGA2ox8* Conferring Osmotic Stress Tolerance in Rice

**DOI:** 10.3390/ijms22179107

**Published:** 2021-08-24

**Authors:** Yinxiao Wang, Fengping Du, Juan Wang, Yingbo Li, Yue Zhang, Xiuqin Zhao, Tianqing Zheng, Zhikang Li, Jianlong Xu, Wensheng Wang, Binying Fu

**Affiliations:** 1 Institute of Crop Sciences/National Key Facility for Crop Gene Resources and Genetic Improvement, Chinese Academy of Agricultural Sciences, South Zhong-Guan-Cun Street 12, Beijing 100081, China; hengshuiyin@163.com (Y.W.); 18643702970@163.com (F.D.); wangjuan22@outlook.com (J.W.); lyb156636865902021@163.com (Y.L.); 15931896028@163.com (Y.Z.); zhaoxiuqin@caas.cn (X.Z.); zhengtianqing@caas.cn (T.Z.); lizhikang@caas.cn (Z.L.); xujianlong@caas.cn (J.X.); 2 Biotechnology Research Institute, Chinese Academy of Agricultural Sciences, South Zhong-Guan-Cun Street 12, Beijing 100081, China; 3 College of Life Sciences, Northwest A&F University, Xianyang 712100, China; 4 College of Agronomy, Anhui Agricultural University, Hefei 230036, China

**Keywords:** gibberellin 2-oxidase gene, osmotic stress tolerance, jasmonate, rice

## Abstract

Gibberellin 2-oxidase (GA2ox) plays an important role in the GA catabolic pathway and the molecular function of the *OsGA2ox* genes in plant abiotic stress tolerance remains largely unknown. In this study, we functionally characterized the rice *gibberellin 2-oxidase 8* (*OsGA2ox8*) gene. The *OsGA2ox8* protein was localized in the nucleus, cell membrane, and cytoplasm, and was induced in response to various abiotic stresses and phytohormones. The overexpression of *OsGA2ox8* significantly enhanced the osmotic stress tolerance of transgenic rice plants by increasing the number of osmotic regulators and antioxidants. *OsGA2ox8* was differentially expressed in the shoots and roots to cope with osmotic stress. The plants overexpressing *OsGA2ox8* showed reduced lengths of shoots and roots at the seedling stage, but no difference in plant height at the heading stage was observed, which may be due to the interaction of OsGA2ox8 and OsGA20ox1, implying a complex feedback regulation between GA biosynthesis and metabolism in rice. Importantly, *OsGA2ox8* was able to indirectly regulate several genes associated with the anthocyanin and flavonoid biosynthetic pathway and the jasmonic acid (JA) and abscisic acid (ABA) biosynthetic pathway, and overexpression of *OsGA2ox8* activated JA signal transduction by inhibiting the expression of jasmonate ZIM domain-containing proteins. These results provide a basis for a future understanding of the networks and respective phenotypic effects associated with *OsGA2ox8*.

## 1. Introduction

Plants are sessile organisms that are constantly exposed to unfavorable environmental stimuli and face the challenge of continuous adaptation to various stresses to maintain growth and development. Rice (*Oryza sativa* L.) is a staple food for half the world’s population, and its yield is strongly restricted by severe drought occurring annually in the majority of rice-growing areas worldwide [1]. Given the environmental deterioration associated with climate change, development of rice cultivars that show high drought tolerance is a matter of urgency to meet the challenging food demands of both the present and the future [2].

Several classes of phytohormones may either act close to or remote from the sites where they are synthesized and may regulate and coordinate the responses of plants to environmental stresses [3]. Gibberellins (GAs) are a class of tetracyclic diterpenoid carboxylic acids. GA_1_, GA_3_, GA_4_, and GA_7_ are bioactive GAs that function as essential growth phytohormones to control a variety of growth and developmental processes throughout the life cycle of plants [4]. Cell division and cell elongation are enhanced by bioactive GAs, which also promote transitions of different developmental stages [5]. The typical GA-deficient phenotypes include dwarfism, inhibited root growth, small dark green leaves, defective flowering, prolonged germination dormancy, male sterility, and reduced seed production [6,7,8,9]. Therefore, it is important for plants to produce and maintain optimal amounts of bioactive GAs to ensure normal growth and development.

Most of the enzymes involved in catalyzing GA biosynthesis and catabolism have been identified [9,10,11]. The GAs are synthesized from trans-geranylgeranyl diphosphate (GGDP) in three major steps, according to the type of enzymes as well as their subcellular localization [12,13]. GGDP is transformed into ent-kaurene in the plastid via the methylerythritol phosphate pathway. Conversion of ent-kaurene to GA_12_ in the endoplasmic reticulum is catalyzed by two membrane-associated P450 mono-oxygenases. GA_12_ is then oxidated in a reaction catalyzed by soluble 2-oxoglutarate-dependent dioxygenases (2-ODDs), GA 20-oxidase (GA20ox), and GA 3-oxidase (GA3ox), in the cytosol [14]. The best-characterized deactivation reaction involves 2β-hydroxylation of bioactive GAs and their precursors, which is catalyzed by GA 2-oxidase (GA2ox). Based on the difference in substrates, GA 2-oxidases can be divided into C_19_-GA2oxs and C_20_-GA2oxs. C_19_-GA2oxs can hydroxylate the C_19_-GA precursors (GA_20_ and GA_9_) or active C_19_-GAs (GA_1_ and GA_4_) to produce biologically inactive GAs [9]. The class C_20_-GA2oxs contains three unique and conserved amino acid motifs that are absent in the C_19_-GA2oxs class [15]. Previous studies have reported that C_20_-GA2oxs, including GA2ox7 and GA2ox8 in *Arabidopsis thaliana* L., GA2ox3 in *Spinacia oleracea* L., and GA2ox6 in *Oryza sativa,* accept C_20_-GAs but not C_19_-GAs as their substrates, and convert GA_12_ and GA_53_ to GA_110_ and GA_97_, respectively [15,16,17].

The expression of certain paralogs within the GA20ox, GA3ox, and GA2ox families provides a mechanism for GA homeostasis [18,19]. Most evidence suggests that the genes encoding dioxygenases act as the main regulatory sites of the GA biosynthetic pathway for developmental and environmental signals, and that *GA2ox* genes particularly participate in response to abiotic stress. *OsGA2ox6* encodes a C_20_-GA2ox, and ectopic expression of *GA2ox6* moderately lowers GA contents and reprograms transcriptional networks, leading to reduced plant height, an increased number of productive tillers, and an expanded root system. In addition, transgenic rice plants ectopically expressing *GA2ox6* show elevated tolerance of drought stress with expanded bulliform cells, higher proline contents, enhanced catalase (CAT) activity, an increased content of ascorbate peroxidase, improved water use efficiency, and an increased photosynthesis rate [20]. *Gossypium hirsutum* L. overexpressing *GhGA2ox1* shows increased tolerance to drought and salt stress, higher contents of free proline and chlorophyll, and increased relative water content compared with those of non-transgenic lines [21]. The overexpression of *StGA2ox1* in *Solanum tuberosum* L. leads to improved salt, drought, exogenous hormone, and low temperature stress tolerance, which may be associated with an enhanced control of chlorophyll, carotenoids, and water loss and the subsequent accumulation of osmoprotectants [22]. Shi et al. [22] believed that *StGA2ox1* can regulate GA synthesis and further affect plant growth and stress responses in potato. The expression of *AaGA2ox1*, *AaGA2ox2*, and *AaGA2ox4* is upregulated under drought or high salt stress in *Artocarpus altilis* (Parkinson) Fosberg [23]. Both *GA2ox7* and *DDF1* (*DWARF AND DELAYED FLOWERING 1*) are induced by high-salinity stress and reduce the amount of bioactive GAs in Arabidopsis [24]. DDF1 binds to DRE-like motifs (GCCGAC and ATCGAC) in the *GA2ox7* promoter to upregulate the expression of *GA2ox7*, which causes GA deficiency and enhances salt tolerance [24]. These results suggest that the GA-dependent growth retardation conferred by GA2ox is an important mechanism for stress adaptation in plants.

Our previous transcriptome analysis revealed that transcript levels of a putative *GA2-oxidase 8* (*OsGA2ox8*, Os05g0560900) were significantly increased in the shoots and decreased in the roots under drought stress, thus showing root-dependent repression [25]. In this study, the functions of OsGA2ox8 in rice were characterized using molecular analysis and plant physiological indices. The expression of *OsGA2ox8* was differentially regulated in the shoots and roots under osmotic stress. Importantly, overexpression of *OsGA2ox8* significantly enhanced osmotic stress tolerance associated with higher contents of osmotic regulators and antioxidants, compared with those of the wild type (WT). Our findings demonstrated that *OsGA2ox8* mediated a multilevel and complex mechanism associated with osmotic stress tolerance.

## 2. Results

### 2.1. Identification and Characterization of OsGA2ox8

**Figure 1:**
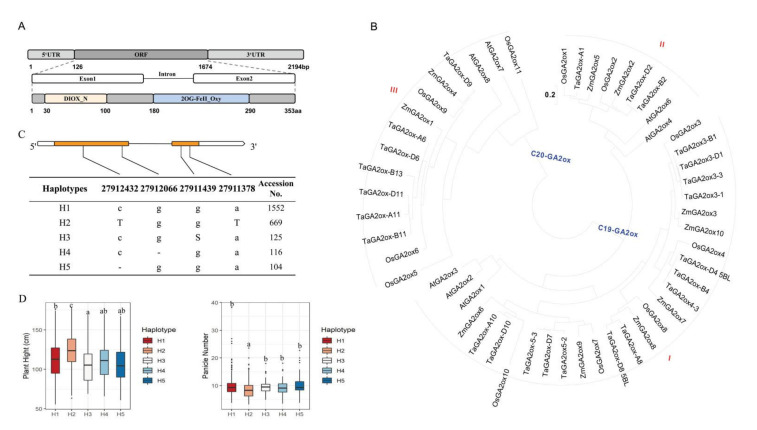
Identification and characterization of *OsGA2ox8*. (**A**) Schematic structure of *OsGA2ox8*. E1 and E2 represent two exons. (**B**) Phylogenetic relationships among GA2ox amino acid sequences from *Triticum aestivum* (Ta), *Oryza sativa* (Os), *Arabidopsis thaliana* (At), and *Zea mays* (Zm). Scale bars indicate number of amino acid substitutions per site. (**C**) Major haplotypes of *OsGA2ox8* coding sequence based on significant single-nucleotide polymorphisms. (**D**) Plant height and panicle number per plant among different haplotypes. Different letters in (**D**) indicate significant differences (*p* < 0.05, one-way ANOVA followed by Tukey’s HSD test) based on data for plant height and panicle number per plant.

*OsGA2ox8* was located on chromosome 5 (http://rapdb.dna.affrc.go.jp/index.html accessed on 16 September 2017) and contained a 1062 bp CDS composed of two exons and one intron (Figure 1A). To investigate the evolutionary relationships of *OsGA2ox8*, we performed a phylogenetic analysis of the amino acid sequences of *OsGA2ox8* together with an additional 51 GA2ox genes from four species, namely *Zea mays* L. (*Zm*), *Oryza sativa* (*Os*), *Triticum aestivum* L. (Ta), and *Arabidopsis* *thaliana* (*At*) (Figure 1B). Consistent with previous studies, *GA2ox* genes were divided into three classes, with the Arabidopsis *GA2ox* genes functioning as node markers [15]. The genes *AtGA2ox1*, *AtGA2ox2*, and *AtGA2ox3* belonged to class I; the genes *AtGA2ox4* and *AtGA2ox6* were grouped in class II; and the genes *AtGA2ox7* and *AtGA2ox8* were grouped in class III. Members of classes I and II catabolize C_19_-GAs, whereas class III members only 2*β*-hydroxylate C_20_-GAs [15]. The phylogenetic analysis showed that *OsGA2ox8* was clustered with *AtGA2ox1*, *AtGA2ox2*, and *AtGA2ox3*, and *OsGA2ox8* belonged to C_19_-GAs (Figure 1B). It has been previously reported that these subgroups of GA2ox proteins might have homologous functions common to monocotyledon and dicotyledon species [16,26].

To understand the genetic variation of *OsGA2ox8* in different materials, haplotypes of the CDS were analyzed using resequencing data for 3K germplasm resources. Four single-nucleotide polymorphisms were detected in the CDS of Os*GA2ox8* that could be divided into five distinct haplotypes, each comprising more than 100 accessions (Figure 1C). Haplotype–phenotype analysis was conducted based on data for grain length, grain width, 1000 grain weight, plant height, and panicle number per plant (http://www.rmbreeding.cn/Genotype/haplotype accessed on 15 March 2019). Significant differences in plant height and panicle number per plant among the haplotypes were observed (Figure 1D), whereas no distinct differences in grain length, grain width, and 1000 grain weight were associated with the individual haplotypes (Supplementary Figure S1).

### 2.2. Characterizing the Expression Profile of OsGA2ox8

**Figure 2:**
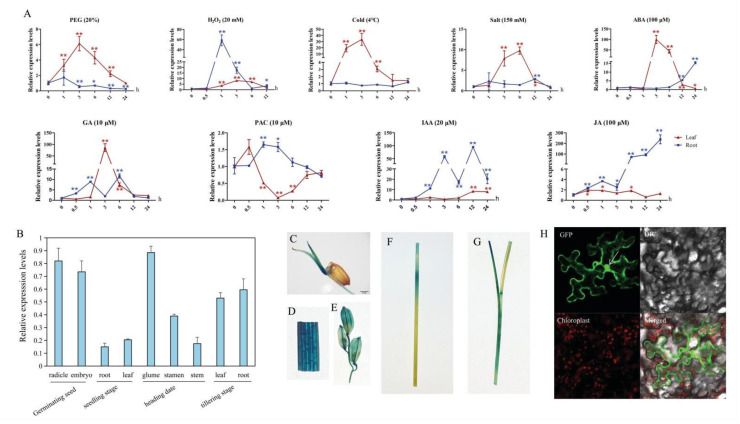
Expression profile of *OsGA2ox8.* (**A**) Expression changes in *OsGA2ox8* in shoots and roots under different treatments analyzed by qRT-PCR. (**B**) Expression levels of *OsGA2ox8* in different tissues at various developmental stages analyzed by qRT-PCR. C–G Histochemical assay of GUS activity driven by the *OsGA2ox8* promoter in the leaves and roots 3 days after germination (**C**), leaf blade (**D**), glume (**E**), stem (**F**), and shoots at the two-leaf stage (**G**). (**H**) Subcellular localization of *OsGA2ox8* in tobacco leaves. Error bars indicate the SD based on three replicates. Asterisks indicate a significant difference compared with 0 h treatment (* *p* < 0.05, ** *p* < 0.01; Student’s *t*-tests).

Previous evidence suggested that *GA2ox* genes act as the main regulatory unit of the GA biosynthetic pathway, participating in response to biotic and abiotic stresses [20,21,22,23]. To investigate whether *OsGA2ox8* is involved in the response to various abiotic stresses and hormones in rice, the expression patterns of *OsGA2ox8* were analyzed using total RNA extracted from the leaves and roots of Nipponbare plants at the three-leaf stage exposed to the following treatments: Gibberellin (GA 10 μM), paclobutrazol (PAC 10 μM), abscisic acid (ABA 100 μM), indoleacetic acid (IAA 20 μM), jasmonic acid (JA 100 μM), hydrogen peroxide (H_2_O_2_ 20 mM), polyethylene glycol 6000 (PEG6000 20%), low temperature (4 °C), and salt (150 mM) (Figure 2A). GA induced the expression of *OsGA2ox8* in the shoots and roots, whereas PAC, acting as an inhibitor of GA, significantly decreased the expression of *OsGA2ox8* in the roots. These results suggested that *OsGA2ox8* participates in the homeostasis of GA. The expression of *OsGA2ox8* was significantly upregulated in shoots and downregulated in roots under PEG treatment, pointing to root-dependent repression. In addition, *OsGA2ox8* showed a similarly high expression in response to the phytohormones ABA, IAA, and JA and to abiotic stresses, including high salt, cold, and H_2_O_2_. Taken together, these results indicated that *OsGA2ox8* also participates in response to various abiotic stresses and hormones. We investigated the expression patterns of 10 additional *OsGA2ox* genes under the salt and PEG treatments (Supplementary Figure S2). The results showed that *OsGA2ox3*, *OsGA2ox5*, *OsGA2ox6*, and *OsGA2ox7* were only induced by salt stress, whereas *OsGA2ox9* and *OsGA2ox10* were induced by both salt stress and PEG treatment, which suggested that *OsGA2ox*s are an important gene family involved in rice adaptation to stresses.

Given that *OsGA2ox8* participated in response to various phytohormones and abiotic stresses, the cis-acting elements in the promoter region (2193 bp upstream of the transcription start site) of *OsGA2ox8* were analyzed using the PLACE database (http://www.dna.affrc.go.jp/PLACE/ accessed on 16 March 2019) (Table 1). The results revealed several *cis*-regulatory elements in the promoter region of *OsGA2ox8*: two ABA-responsive element-like elements (ABRELATERD1 and ABRERATCAL) [27]; two DRE-related elements (DRE2COREZMRAB17 and DRECRTCOREAT) recognized by AP2/ERF proteins [28,29]; three copies of JA-responsive CGTCA motifs; one CBFHV motif, which is a dehydration-responsive element [30]; one GA-responsive element (GAREAT) [31]; five copies of the ATCTA motif identified as an ERF-binding element [32]; and two low-temperature responsive elements (LTRECOREATCOR15 and LTRE1HVBLT49) [33,34]. All the aforementioned cis-elements are potentially involved in the *OsGA2ox8*-mediated response to environmental stress and hormone signaling.

To more clearly define the function of the *OsGA2ox8* promoter, a series of promoter fragments of varying lengths, comprising 2193 bp, 1301 bp, and 647 bp, were cloned into the pMDC162 vector in which *GUS* was driven by the inserted fragments for transient transformation of rice protoplasts and genetic transformation. The GUS activity in rice protoplasts or transgenic plants transformed with the *OsGA2ox8*-pro1 (2193 bp) and *OsGA2ox8*-pro2 (1301 bp) vectors was significantly higher than that induced by *OsGA2ox8*-pro3 (647 bp) (Supplementary Figure S3). These results indicated that the 2193 bp and 1301 bp upstream fragments contained promoter activity and were able to drive the expression of *OsGA2ox8.*

To investigate the expression profiles in different tissues at different developmental stages, qRT-PCR analyses (Figure 2B) and histochemical staining of GUS activity using *OsGA2ox8*-Pro1::GUS transgenic plants was performed (Figure 2C–G). The results revealed that *OsGA2ox8* was expressed at various stages but showed relatively higher expression in the radicle and embryo of germinating seeds, the glume at the heading stage, and the leaf and root at the tillering stage.

The full-length open reading frame of *OsGA2ox8* was fused to GFP and transformed into tobacco leaves. Fluorescence was ubiquitously observed in the nucleus, cytoplasm, and plasma membrane (Figure 2H), which indicated that OsGA2ox8 might be involved in a diverse range of biological processes.

### 2.3. Identification of the Osmotic Stress Tolerance Phenotype and Physiological Index Determination of OsGA2ox8

To determine the biological function(s) of *OsGA2ox8* in response to osmotic stress, we constructed *OsGA2ox8* overexpression (OE) lines and CRISPR/Cas9 knockout (KO) mutants. Five T2 generation OE lines (OE-1 to -5) and four T2 generation KO mutants (KO-1 to -4) were obtained. Sequencing analysis showed the presence of a 2 bp deletion and a T/A insertion at different positions of the CDS region in KO-1, KO-4, and KO-3 that resulted in frame-shift mutations in *OsGA2ox8.* A 1 bp deletion at position 118 of the CDS in KO-2 led to a premature translation termination codon and thus resulted in a null mutation (Supplementary Figure S4A). The qRT-PCR results showed that the expression of *OsGA2ox8* was upregulated in OE lines and downregulated in KO lines to various extents, compared with that of the WT under the non-stress treatment (Supplementary Figure S4B). Based on the qRT-PCR results and sequencing analysis, three OE lines (OE-2, OE-3, and OE-4) and three mutant lines (KO-1, KO-2, and KO-3) were used to investigate phenotypic performance under osmotic stress at the seedling stage.

**Figure 3:**
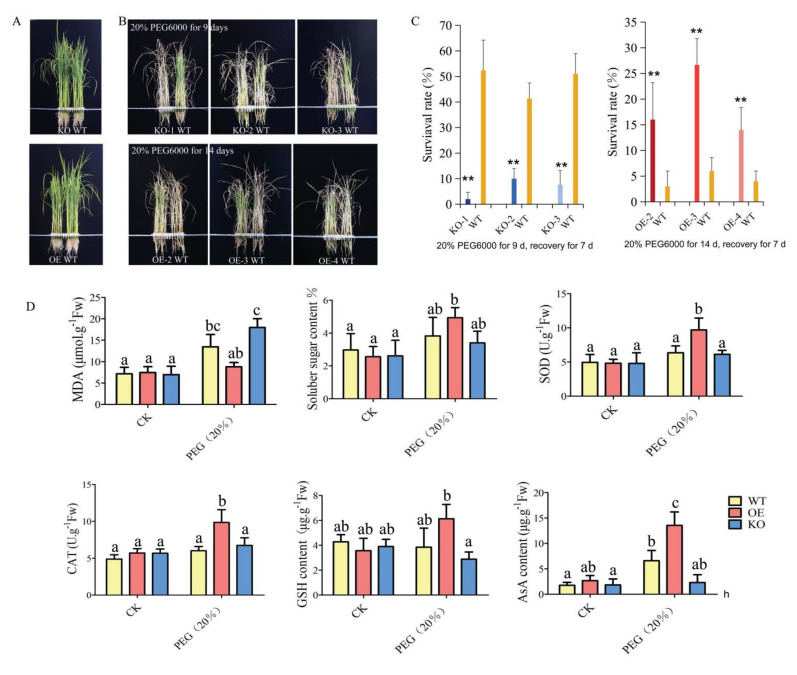
Overexpression of *OsGA2ox8* enhanced osmotic stress tolerance of rice at the seedling stage. (**A**) Phenotypes of the *OsGA2ox8* transgenic lines and wild-type plants under the non-stress condition. (**B**) Phenotypes of the *OsGA2ox8* transgenic lines and wild-type plant treated with 20% PEG6000 (~1.1 MPa) for 9 and 14 days, respectively, at the seedling stage. (**C**) Survival frequencies of transgenic lines and wild-type plants treated with 20% PEG6000 for 9 and 14 days, respectively, and then subjected to recovery for 7 days. (**D**) Physiological changes in *OsGA2ox8* transgenic lines and wild-type plants under the non-stress condition and 20% PEG6000 treatment. Error bars in C indicate the SD based on data for three replicates. Asterisks indicate a significant difference compared with the wild type (** *p* < 0.01; Student’s *t*-tests). Different letters indicate significant differences (*p* < 0.05, one-way ANOVA followed by Tukey’s HSD test) based on data for three replicates.

The plants overexpressing *OsGA2ox8* showed significantly reduced shoot length, whereas the shoot length of KO mutants increased under the non-stress condition (Figure 3A). The OE and KO plants exhibited different sensitivities in response to osmotic stress (Figure 3B). On evaluating morphological changes, the leaves of KO lines first appeared curly after treatment with 20% PEG6000 (~1.1 MPa) and wilted after osmotic stress, whereas the OE lines were more resilient than the KO lines and WT to osmotic stress for 9 days and showed superior recovery 7 days after the conclusion of osmotic stress. All three transgenic OE lines exhibited improved stress tolerance compared with the WT, as indicated by the significantly higher frequencies of seedling survival. In contrast, the three KO lines were more sensitive to osmotic stress and showed significantly decreased survival frequencies compared with the WT after rewatering for 7 days (Figure 3C). These results suggested that the expression of *OsGA2ox8* was positively involved in osmotic stress tolerance. Hence, we selected the transgenic lines OE-2 and KO-2 to characterize the functionality of *OsGA2ox8* in additional experiments.

Physiological traits of OE-2, KO-2, and WT under the control and osmotic stress treatment were compared. The OE-2 plants showed the lowest malondialdehyde (MDA) content and the highest amounts of soluble sugar, glutathione (GSH), and ascorbic acid (AsA), as well as the highest activities of superoxide dismutase (SOD) and catalase (CAT) during osmotic stress. In contrast, we observed the opposite pattern in the KO-2 plants (Figure 3D). These results strongly suggested that the overexpression of *OsGA2ox8* improved osmotic stress tolerance in rice by maintaining the stability of the membrane system and by enhancing osmotic adjustment and antioxidant activity.

### 2.4. OsGA2ox8 Was Differentially Expressed in the Shoots and Roots under Osmotic Stress

**Figure 4:**
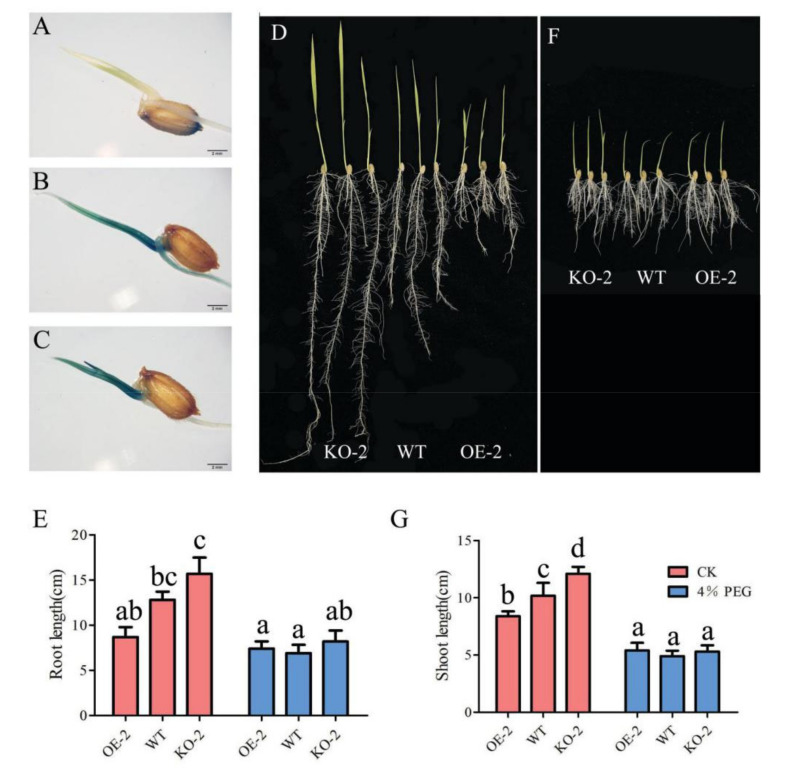
*OsGA2ox8* was differentially expressed in shoots and roots under osmotic stress. (**A**–**C**) Histochemical assay of GUS activity driven by the *OsGA2ox8* promoter (2193 bp) under the non-stress condition (**B**) and osmotic stress (**C**), as well as the wild-type plants (**A**). (**D**–**G**) Shoot and root length of transgenic lines and the wild type at 10 days after germination under the non-stress condition (**D**,**E**) and osmotic stress (**F**,**G**). Different letters in (**E**) and (**G**) indicate significant differences (*p* < 0.05, one-way ANOVA followed by Tukey’s HSD test) based on data for three replicates.

Our previous transcriptome analysis revealed that the expression of *OsGA2ox8* showed root-dependent repression under drought stress [25]. To detect dynamic changes in *OsGA2ox8* expression under osmotic stress, a GUS staining experiment with *OsGA2ox8*-pro1 transgenic plants was conducted. GUS staining showed that *OsGA2ox8* was strongly expressed in the roots and leaves at 3 days’ post-germination, and that the highest expression level was detected in vascular tissue (Figure 4B). After treatment of the seeds with 4% PEG6000 for 3 days, the expression of *OsGA2ox8* in the roots was significantly decreased and no GUS staining was apparent (Figure 4C).

We evaluated the progression of shoot and root growth under osmotic stress in OE-2, KO-2, and WT plants. The results showed that the overexpression of *OsGA2ox8* decreased the length of shoots and roots, and knockout of *OsGA2ox8* increased the length of shoots and roots at the seedling stage under the non-stress treatment (Figure 4D,E,G). The shoots and roots of all transgenic lines and WT showed no obvious difference under osmotic stress (Figure 4E–G). These results indicated that *OsGA2ox8* was differentially expressed in the shoots and roots under osmotic stress and that root elongation was enhanced under osmotic stress, allowing the roots to penetrate deeper under the stress treatment.

### 2.5. OsGA2ox8 Decreased the Length of Shoots and Roots at the Seedling Stage

The roots and shoots of KO-2 plants were significantly elongated, and roots and shoots of OE-2 plants were shortened, compared with those of the WT under non-stress condition (Figure 3A and Figure 4D). Moreover, the OE and KO lines showed no difference in plant height, tiller number, 1000 grain weight, and grain length at maturity compared with those of the WT, although the KO lines showed higher grain width (Supplementary Figure S5).

**Figure 5:**
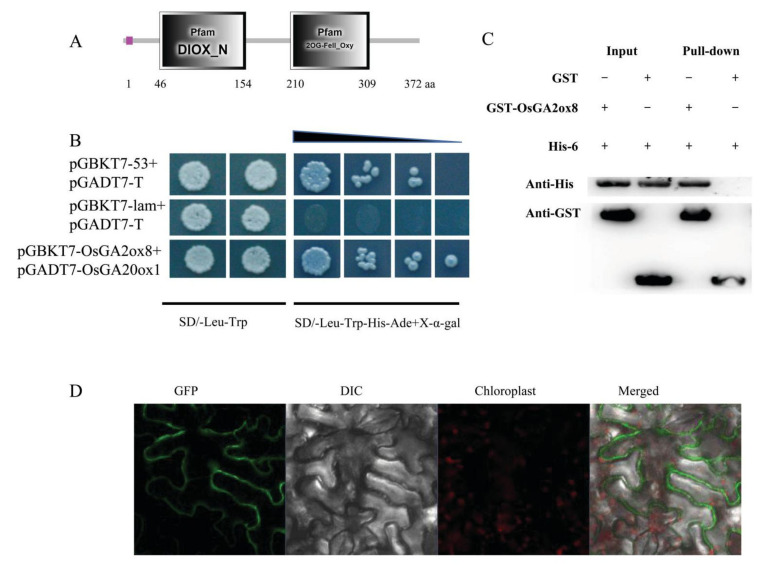
OsGA2ox8 interaction with OsGA20ox1. OsGA20ox1 contained two conserved domains: the non-heme dioxygenase domain and the 2OG-Fe (II) oxygenase superfamily domain (**A**). OsGA2ox8 interacted with OsGA20ox1 in a yeast two-hybrid assay (**B**), pull-down assay (**C**), and BiFC assay in *Nicotiana benthamiana* leaves (**D**). pGADT7-T was used as a positive control prey plasmid; pGBKT7-53 and pGBKT7-lam were used as positive and negative control bait plasmids, respectively. pnYFP-OsGA2ox8 and pcCFP-OsGA20ox1 were used in the BiFC assay.

Gibberellin promotes elongation of the root and stem, and the expression of *GA2ox* gene families are involved in GA metabolism [18,19]. To detect the GA metabolism regulatory mechanism of *OsGA2ox8* at the different growth stages of rice, a yeast two-hybrid assay was performed to identify the proteins that interacted with OsGA2ox8. Twelve interacting proteins from 65 positive clones were screened and are listed in Supplementary Table S1. Results from BiFC and pull-down assays revealed a direct interaction between OsGA20ox2 and OsGA2ox8 (Figure 5). OsGA20ox2 and OsGA2ox8 are involved in catalyzing GA biosynthesis and catabolism, respectively [35]. Thus, the interaction between the two genes indicated that a more complex regulatory mechanism might control the homeostasis of GA.

### 2.6. Transcriptome Analysis of the Transgenic and WT Plants under the Non-Stress Condition

To investigate the reproducibility of the biological replicates and the relationship between the different lines, a correlation analysis was performed based on the global patterns of gene expression. High reproducibility between the biological replicates was evident (Supplementary Figure S6A). The 18 samples were divided into two main groups; those grown under the non-stress condition clearly diverged from those subjected to osmotic stress. Moreover, independent of the growing condition, the three replicates of each line clustered together, which supported the difference in osmotic stress tolerance of the OE and KO lines.

To gain insights into the genes and pathways involved in osmotic stress tolerance regulated by *OsGA2ox8*, the DEGs were identified under the criteria of false discovery rate < 0.01 and |log_2_ fold change| > 1 (Supplementary Figure S6B). A qRT-PCR analysis was performed to validate the accuracy of the RNA-seq data, and 21 DEGs associated with multiple functions were selected randomly (Supplementary Table S2). A list of the primers used is presented in Supplementary Table S3. A strong correlation (*R*^2^ = 0.877) between the qRT-PCR results and the transcriptome data was observed, demonstrating the accuracy and reliability of the latter data (Supplementary Figure S6C).

In comparison with the WT plants, we detected a total of 509 upregulated and 376 downregulated genes in the OE-2 line under the non-stress condition, whereas 866 and 338 genes, respectively, in the KO-2 line were observed (Supplementary Figure S6B). Of these DEGs, 279 upregulated genes and 135 downregulated genes overlapped between the two genotypes (Supplementary Figure S7A). The proportion of DEGs that were associated with GA, ABA, JA, IAA, and salicylic acid biosynthesis was significantly higher in KO-2 than OE-2 (compared with the WT) plants under the non-stress condition (Supplementary Figure S7B,C). We then analyzed the genes associated with GA, ABA, and JA synthesis and metabolism (Supplementary Figure S7D). With the exception of *OsGA2ox8*, we observed that an additional four *OsGA2ox* genes were differentially expressed between the transgenic and WT plants under the non-stress condition, which may be associated with the fact that *OsGA2ox8* reduces the amount of GAs at the seedling stage. Moreover, four genes that participate in JA synthesis and two genes associated with the synthesis and metabolism of ABA also showed differential expression. These results indicated that the differential expression levels of *OsGA2ox8* between transgenic and WT plants are able to change multiple physiological and biochemical processes under the non-stress condition in rice.

### 2.7. Transcriptome Analysis of the Transgenic Lines and WT under Osmotic Stress

**Figure 6:**
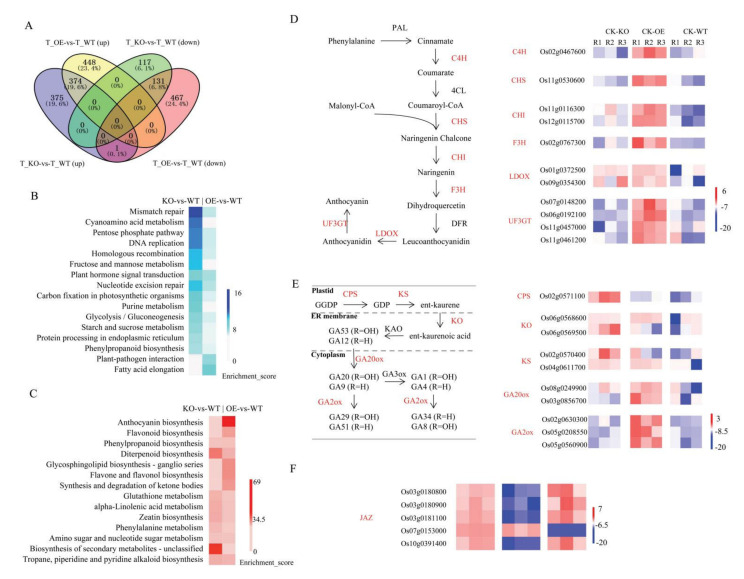
Differentially expressed genes (DEGs) among the transgenic lines and wild-type plants under osmotic stress. (**A**) Overlap of up and downregulated DEGs between *OsGA2ox8* overexpression and CRISPR/Cas9 knockout lines compared with the wild type under osmotic stress. (**B**) KEGG pathway enrichment of the uniquely downregulated genes in the KO-2 and OE-2 lines, compared with wild-type plants. (**C**) KEGG pathway enrichment of the uniquely upregulated genes in the KO-2 and OE-2 lines compared with wild-type plants. (**D**) Diagram of the anthocyanin biosynthetic pathway in *Arabidopsis* and the expression levels of DEGs participating in the anthocyanin biosynthetic process. C4H: cinnamate 4-hydroxylase, PAL: phenylalanine ammonia lyase, CHI: chalcone isomerase, 4CL: 4-coumaroyl:CoA-ligase, DFR: dihydroflavonol reductase, CHS: chalcone synthase, F3H: flavanone 3-hydroxylase, LDOX: leucoanthocyanidin dioxygenase, UF3GT: UDP-Glc:flavonoid 3-O-glucosyltransferase. (**E**) Diagram of the GA biosynthetic pathway and the expression levels of DEGs participating in the GA biosynthetic process. GGDP: trans-geranylgeranyl diphosphate, KO: ent-kaurene oxidase, KS: ent-kaurene synthase, CPS: ent-copalyl diphosphate synthase, GA20ox: gibberellin 20 oxidase, KAO: ent-kaurenoic acid oxidase, GA3ox: gibberellin 3 beta-hydroxylase, GA2ox: gibberellin 2 oxidase. (**F**) Expression levels of jasmonate ZIM domain-containing proteins under osmotic stress. The heatmap presents normalized log_2_ FPKM expression values.

Genes conferring stress resistance that were associated with *OsGA2ox8* in rice were further investigated by comparing DEGs between transgenic lines and WT plants under osmotic stress. Using the threshold of a two-fold change in expression level, 822 upregulated and 599 downregulated genes were detected in the OE-2 line compared with the WT, whereas 750 and 248 genes, respectively, were detected in the KO-2 line (Supplementary Figure S6B). Of these DEGs, 374 upregulated and 131 downregulated genes overlapped between KO-2 and OE-2 (both compared with the WT) under osmotic stress (Figure 6A). A KEGG enrichment analysis showed that the genes that were particularly downregulated in OE-2 were enriched in plant hormone signal transduction, fatty acid elongation, and plant–pathogen interaction. In contrast, the genes that were uniquely downregulated in KO-2 were enriched in multiple pathways, such as cyanoamino acid metabolism, starch and sucrose metabolism, and plant hormone signal transduction (Figure 6B). Furthermore, genes participating in diterpenoid biosynthesis and in arginine, proline, and glutathione metabolism were upregulated in KO-2, whereas genes that were associated with anthocyanin biosynthesis and flavonoid biosynthesis were remarkably increased in OE-2 (Figure 6C).

The DEGs associated with anthocyanin and flavonoid biosynthesis were remarkably enriched in OE-2 under osmotic stress. Further analysis showed that many DEGs associated with anthocyanin biosynthesis were upregulated in OE-2 under osmotic stress (Figure 6D). Os06g0192100 and Os01g0372500 encode anthocyanidin 3-O-glucosyltransferase (UF3GT) and leucoanthocyanidin dioxygenase 1 (LDOX), respectively, which are crucial enzymes in the biosynthesis of anthocyanin [36]. Both Os12g0115700 and Os11g0116300 encode chalcone–flavanone isomerase (CHI), which might increase the accumulation of proanthocyanidin and flavonol in Arabidopsis [37]. Moreover, Os10g0317900 and Os04g0101400, two cytochrome P450 genes, impact on the hydroxylation pattern of anthocyanidins (the chromophores and precursors of anthocyanins) and play an important role in the biosynthesis of flavonoids and anthocyanins [38]. In addition, the arginine and proline metabolic pathway was enriched among the upregulated genes in the KO-2 line. Polyamines, putrescine, and proline are osmoprotectants and important mediators of abiotic stress tolerance. Os09g0368200, which encodes a polyamine oxidase, catalyzes the oxidation of spermine and polyamines spermidine, contributing to the accumulation of H_2_O_2_ [39]. Os04g0107600 and Os09g0543400 encode arginine decarboxylase 2 and ornithine decarboxylase, respectively, and are able to modulate the amount of polyamine [40]. Glutathione, carotenoids, anthocyanins, and flavonoids are important antioxidants and might help to eliminate the excessive active oxygen that is produced under abiotic stresses [41,42,43,44,45]. Unsaturated fatty acids, fatty acids, and proline act as important osmoprotectants in response to drought stress [41,43,44,46]. The observed changes in antioxidants and osmoprotectants may endow the OE line with superior antioxidant and osmotic adjustment capacity.

The genes participating in plant hormone signal transduction (especially of JA and ABA), the biosynthesis of diterpenoids (a substrate for GA synthesis), and the metabolism of phenylalanine (biosynthesis of secondary metabolites) were enriched in the KO-2 and OE-2 lines compared with WT plants (Figure 6B,C), which may indicate the involvement of a complex network of multiple hormones. Based on the comparative KEGG enrichment analysis, we decided to focus on the analysis of DEGs that were associated with the biosynthesis of GA, ABA, and JA, as well as their signal transduction. Thus, the expression pattern of genes involved in the GA metabolism pathway under osmotic stress was investigated. Bioactive GAs, such as GA_1_ and GA_4_, are synthesized from trans-geranylgeranyl diphosphate (GGDP), as shown in Figure 6E. Ent-copalyl diphosphate synthase (CPS), ent-kaurene synthase (KS), and ent-kaurene oxidase (KO) are the crucial enzymes catalyzing the early steps in the GA biosynthetic pathway. One CPS gene (*OsCPS2*), one KO gene (*OsKO4*), and two KS genes (*OsKS7* and *OsKS3*) were increased in the KO-2 line, whereas only one KO gene (*OsKO5*) and one KS gene (*OsKS3*) were upregulated in OE-2 under osmotic stress. The GA20ox and GA3ox enzymes catalyzed later steps in the GA biosynthetic pathway, and two *OsGA20ox* genes showed changes in expression levels under osmotic stress. Furthermore, with the exception of *OsGA2ox8*, the genes Os02g0630300 (*OsGA2ox9*) and Os05g0208550 (*OsGA2ox10*) were also upregulated in OE-2 but showed no expression changes in KO-2 under osmotic stress. The aforementioned changes in expression levels may indicate a complex regulatory mechanism of GA in KO and OE lines.

KEGG enrichment showed that DEGs were enriched in the JA and ABA biosynthetic pathway and JA signal transduction. The enzymes aldehyde oxidase (AAO) and 9-*cis*-epoxycarotenoid dioxygenase (NCED) play a crucial role in the ABA biosynthesis [47]. Under osmotic stress, we detected one gene encoding NCED (Os02g0704000) and one encoding AAO (Os03g0798101) that were upregulated in OE-2, whereas no NCED or AAO showed changes in their respective levels of expression in KO-2, which may contribute to the higher amount of ABA in the OE-2 line., Three distinct pathways, including the octadecane pathway and the hexadecane pathway, are responsible for the synthesis of Jas in *Arabidopsis* [48]. Lipoxygenase (LOX) is a vital enzyme in JA biosynthesis. Two genes encoding LOX (Os02g0194700 and Os05g0304600) showed changes in expression under osmotic stress. Specifically, both LOX genes were increased in OE, but only Os02g0194700 was upregulated in KO-2. Jasmonate ZIM domain-containing proteins (JAZ) actively repress JA signal transduction by interacting with JA-responsive transcription factors. Interestingly, we detected five JAZ genes showing changes in expression under osmotic stress. Of these genes, four were decreased in OE and one was upregulated in both OE and KO compared with the WT plants (Figure 6F). The DEGs associated with JA synthesis and signal transduction suggested that the higher JA content is associated with activated JA signal transduction in the OE-2 line, compared with both WT and KO plants.

### 2.8. Metabolic Characteristics Response to Osmotic Stress

**Figure 7:**
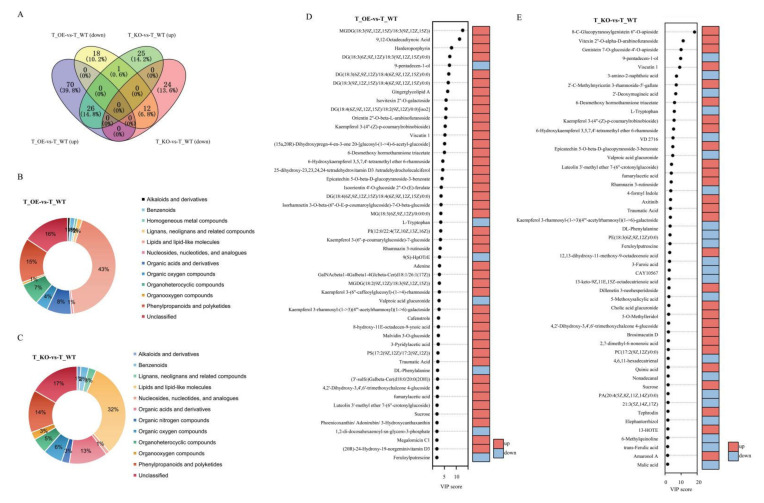
Important differentially expressed metabolites (DEM) identified by partial least squares–discriminant analysis (PLS–DA) in overexpression and CRISPR/Cas9 knockout lines, compared with wild-type plants. (**A**) Overlap of up and downregulated DEMs between *OsGA2ox8* overexpression and CRISPR/Cas9 knockout lines compared with wild-type plants under osmotic stress. (**B**) Classification of DEMs between an *OsGA2ox8* overexpression line and the wild type. (**C**) Classification of DEMs between an *OsGA2ox8* CRISPR/Cas9 knockout line and the wild type. (**D**) Fifty top DEMs according to the variable importance in projection (VIP) scores in an *OsGA2ox8* overexpression line compared with the wild type. (**E**) Fifty top DEMs according to the VIP score in a CRISPR/Cas9 knockout line compared with the wild type.

A global untargeted metabolite analysis based on ESI positive and ESI negative ion modes was conducted to identify the metabolic response of rice seedling to PEG stress. We performed OPLS-DA to visualize the metabolic changes among transgenic and WT plants (Supplementary Figure S8). Based on the thresholds VIP > 1 and *p*-value < 0.05, we identified, under osmotic stress, 127 and 88 differentially expressed metabolites (DEMs) with important variation in OE-2 (Supplementary Table S4) and KO-2 (Supplementary Table S5), respectively. Of these DEMs, 26 upregulated and 12 downregulated metabolites overlapped between KO and OE under osmotic stress (Figure 7A). The classification of the DEMs in OE-2 and KO-2 is shown in Figure 7B,C. The top 50 DEMs in OE-2 and KO-2, as well as their changing patterns, are presented in Figure 7D,E. Compared with the KO line, a higher number of metabolites accumulated in the OE line under osmotic stress, including sucrose, flavonoids, fatty acids and conjugates, linoleic acids and derivatives, and steroid conjugates. Sucrose might be accumulated either by starch degradation or reduction in sugar transfer from mesophyll cells to the phloem, which would indicate that growth restriction resulted from the stress [46]. Sucrose is known to be an osmoprotectant in response to drought and salt stress [49], while flavonoids and anthocyanins are important secondary metabolites that might act as scavengers of vacuolar reactive oxygen species during osmotic stress [44]. We observed an accumulation of 27 flavonoids and a decrease in only two flavonoids in OE plants, which may confer this line with a high antioxidant capacity. We also noted an increase in the amount of unsaturated fatty acids and conjugates, such as traumatic acid, fumarylacetic acid, 7-methoxy-9-methyl-4*E*, 8*E*-hexadecadienoic acid, and 9,12-octadecadiynoic acid in the OE-2 line. Such an increase in the amount of unsaturated fatty acids can significantly improve the tolerance of *Arabidopsis thaliana* to low temperatures and oxidative stress [50]. Moreover, the ratio of malic acid to a fumaric acid in guard cells plays an important role in regulating stomatal opening [51]. We observed a significant decrease in the amount of L-phenylalanine and D-tryptophan in the OE line compared with the WT. These two compounds belong to the group of aromatic amino acids (AAAs) and might serve as precursors for large numbers of secondary metabolites and phytohormones [44]. Hence, the decrease in the amount of L-phenylalanine and D-tryptophan may result from an increase in the number of secondary metabolites, such as flavonoids and anthocyanins. Taken together, these results indicated that changes in the metabolite profiles may result in differences in tolerance to osmotic stress tolerance between OE-2 and KO-2 plants.

## 3. Discussion

Eleven *GA2ox* genes have been identified in the rice genome [52]. Most evidence suggests these genes are the main regulatory sites of the GA metabolism pathway and are particularly responsive to biotic/abiotic stress [20,21,23,24]. Abiotic stresses, including water deficit, salt stress, and extreme temperature, resulted in the accumulation of reactive oxygen species, which led to oxidative damage in plant cells [53]. The present experiments showed that, during osmotic stress, rice plants overexpressing *OsGA2ox8* showed lower contents of MDA and higher contents of soluble sugars, GSH, and AsA, as well as higher activities of SOD and CAT relative to those of the WT. These modifications were the opposite in the case of KO-2 plants (Figure 3). Our transcriptome and metabolome analyses of the transgenic and WT plants showed a significant increase in DEGs associated with anthocyanin biosynthesis and flavonoid biosynthesis in the OE line relative to that of the WT. In contrast, DEGs participating in the biosynthesis of unsaturated fatty acids, carotenoids, and fatty acids were significantly reduced in the KO lines. Furthermore, some metabolites, including sucrose, flavonoids, choline, and porphyrins, were highly accumulated only in the OE-2 line under osmotic stress. Importantly, flavonoids, anthocyanins, GSH, AsA, SOD, and CAT might act as scavengers of vacuolar reactive oxygen species during osmotic stress and, similar to proline, sucrose is known to act as an osmoprotectant [46,49]. Compared with the WT plants, the OE-2 line showed decreased abundances of L-phenylalanine, L-asparagine, and D-tryptophan, which are among the group of aromatic amino acids (AAAs) that might serve as precursors for large numbers of secondary metabolites [44]. The decrease in contents of L-phenylalanine and D-tryptophan may result from an increase in the number of secondary metabolites, such as flavonoids and anthocyanins. These results strongly suggested that overexpression of *OsGA2ox8* improved tolerance to osmotic stress in rice by maintaining the stability of the membrane system, and improving and enhancing osmotic adjustment and antioxidant activities.

A number of cis-regulatory elements associated with phytohormones are present in the promoter region of *OsGA2ox8*, including two ABA-responsive element-like elements, two DRE-related elements, three copies of a JA-responsive motif, and one GA-responsive element. All these cis-elements are potentially involved in the *OsGA2ox8*-mediated response to environmental stress and various hormone signaling pathways. *OsGA2ox8* was upregulated in response to treatment with exogenous ABA, IAA, and JA, and abiotic stress treatments, such as high salinity, low temperature, and H_2_O_2_. Transcriptome analysis of transgenic and WT plants under osmotic stress showed that many genes associated with phytohormones were differentially expressed in the OE-2 line, including the auxin biosynthesis and signaling genes Os01g0785400, Os02g0769100, and Os02g0643800 [54,55], the JA-responsive transcription factors Os03g0181100, Os03g0180800, Os10g0391400, and Os03g0180900 [56,57], the ABA biosynthesis gene *OsABA8ox1* *(*Os02g0703600) [58], and the ABA signaling gene *OsABI5* *(*Os01g0859300) [59]. Taken together, these results suggested that *OsGA2ox8* likely participated in the response to various abiotic stresses and hormones, and that coordination between maintaining growth and resisting stress was superior in the OE lines in response to osmotic stress.

Our previous transcriptome analysis revealed that the expression of *OsGA2ox8* showed an opposite trend in the shoots and roots of rice under drought stress [25]. In the current study, we performed GUS staining and showed that the expression of *OsGA2ox8* was significantly decreased in the roots, and that no GUS staining was detected when the seeds were treated with 4% PEG6000 for 3 days. Overexpression of *OsGA2ox8* decreased the length of shoots and roots, and knockout of *OsGA2ox8* increased the length of shoots and roots at the seedling stage under the non-stress condition (Figure 4D,E,G). The shoots and roots of all transgenic lines and the WT showed no obvious difference under osmotic stress (Figure 4E–G). From these results, we hypothesize that the differential expression of *OsGA2ox8* may result in the differential regulation of responses to osmotic stress in the shoots and roots. *OsGA2ox8* was significantly decreased in the roots under osmotic stress. The expression of *OsGA2ox8* was significantly increased in the shoots under osmotic stress, leading to the inhibition of shoot growth, which might help the plant to conserve energy and improve resistance to osmotic stress.

Semi-dwarfism is a major factor in increasing the yield of green revolution varieties that exhibit an enhanced photosynthesis rate and resistance to damage by wind and rain [60,61]. The best-characterized deactivation reaction for bioactive GAs is catalyzed by GA2oxs. It is generally true that the overexpression of C_20_ GA2oxs causes less severe GA-defective phenotypes in rice than does the overexpression of C_19_ GA2oxs. For example, constitutive ectopic overexpression of C_19_ GA2oxs, such as GA2ox1 and GA2ox3, causes severe dwarfism and absence of seeds in rice, despite the long cultivation periods [26,62]. These responses mainly occur because active GAs are probably deactivated as soon as they are produced [16,63]. In the present study, the overexpression of *OsGA2ox8*, which belongs to the C_19_ GA2ox class, offers two alternative approaches for breeding plants with increased grain width and enhanced osmotic stress tolerance. Unlike other C_19_ GA2oxs, such as GA2ox1 and GA2ox3, *OsGA2ox8* showed a diminished ability to reduce plant height and interacted with the gibberellin biosynthesis gene *OsGA20ox2*. A complex association between *OsGA20ox2* and *OsGA2ox8* may contribute to the regulation of gibberellin biosynthesis and metabolism. The present results provide important information for future applications of *OsGA2ox8* to improve rice yields.

## 4. Conclusions

In the current study, we identified and characterized the C_19_ GA2ox gene *OsGA2ox8*. We observed that *OsGA2ox8* was induced by abiotic stress, including drought, low temperature, and salt. Transgenic rice plants overexpressing *OsGA2ox8* exhibited enhanced osmotic stress tolerance with higher survival frequencies relative to the WT, whereas KO plants showed increased osmotic stress sensitivity with lower survival frequencies in comparison with the WT. In combination, these results demonstrated that *OsGA2ox8* played a crucial positive role in the rice response and tolerance to osmotic stress.

## 5. Materials and Methods

### 5.1. Plant Materials and Growth Conditions

*Oryza sativa* ‘Nipponbare’ was used as the WT for all analyses. For the seedling analysis, we soaked the dried seeds in 0.3% sodium hypochlorite for 1 day and then in water for approximately 3 days at 28 °C until germination. All plants were cultivated with the following settings: 28 °C/26 °C (day/night), a 16 h/8 h (light/dark) photoperiod, and a light intensity of 600 μmol m^−2^ s^−1^. Yoshida nutrient solution was used as the nutrient source.

### 5.2. Stress and Phytohormone Treatments

To evaluate the stress tolerance phenotype, rice seedlings, comprising three overexpression lines (OE), three CRISPR/Cas9 knockout (KO) mutant lines, and the WT, were hydroponically cultured in Yoshida nutrient solution and treated with 20% polyethylene glycol 6000 (PEG6000) (~1.1 MPa) for approximately 7 days, depending on plant sensitivity, until a response to osmotic stress was observed. Thereafter, the seedlings were cultured in Yoshida nutrient solution without PEG6000 for recovery, and seedlings that developed new leaves were counted as survivors. The survival rate was calculated by the formula that the number of survivors was divided by the total number of seedlings. To determine the expression levels of *OsGA2ox8* under different phytohormone and stress treatments, the germinated seeds were cultured in a 96-well bottomless PCR plate in Yoshida nutrient solution. Seedlings at the three-leaf stage were treated with either 10 μM gibberellin (GA), 10 μM paclobutrazol (PAC), 100 μM abscisic acid (ABA), 20 μM indoleacetic acid (IAA), 100 μM jasmonic acid (JA), 20 mM hydrogen peroxide (H_2_O_2_), 20% PEG6000, 120 mM NaCl, or low temperature (4 °C). Shoots and roots were sampled at 0 h, 1 h, 3 h, 6 h, 12 h, and 24 h after initiation of treatment and stored at −80 °C for analysis of *OsGA2ox8* expression.

### 5.3. RNA Extraction and qRT-PCR Analysis

Total RNA was extracted from leaves or roots using Direct-zol™ RNA MiniPrep Kits (Zymo Research, Orange County, CA, USA) and TRIzol Reagent (Life Technologies, Carlsbad, CA, USA) following the manufacturer’s instructions. The synthesis of first-strand cDNA was performed with FastKing gDNA Dispelling RT SuperMix (Tiangen Biotech, Beijing, China). Quantitative real time PCR (qRT-PCR) was conducted using SYBR^®^ Premix Ex Taq™ II (TaKaRa, Kyoto, Japan), in accordance with the manufacturer’s instructions, on an Applied Biosystems^®^ 7500 thermocycler (Thermo Fisher Scientific, Waltham, MA, USA). The relative expression level of each gene was calculated according to the 2^−^^△△*C*t^ method [64]. Error bars indicate the standard deviation (SD) based on three technical replicates (*n* = 6). *OsUBQ5* was used as an internal control to normalize expression of the target genes. The primers used in the qRT-PCR experiment are listed in Supplementary Table S3.

### 5.4. Vector Construction and Rice Genetic Transformation

The full-length transcript of *OsGA2ox8* was obtained from the cDNA library using gene-specific primers, and then cloned into the pCUbi1390 vector in which *OsGA2ox8* was driven by the *Ubi* promoter to generate an *OsGA2ox8* overexpression vector. One target sequence (GGCGCGGCGGCGGCGGTGGC) was selected to generate *OsGA2ox8* knockout mutants using the CRISPR/Cas9 gene-editing system with the binary vector pYLCRISPR/Cas9P_ubi_-H in accordance with the method described by Ma et al. [65]. A fragment of approximately 2.2 kb upstream of the translation start site of *OsGA2ox8* was amplified from the rice genomic DNA. Three promoter fragments of different lengths, comprising 2193 bp, 1301 bp, and 647 bp, were cloned into the pMDC162 vector, using the Gateway™ system, in which β-glucuronidase (*GUS*) was driven by the inserted fragments. All constructed vectors were introduced into Nipponbare via *Agrobacterium*-mediated transformation. The primers used in the experiment are listed in Supplementary Table S3.

### 5.5. Sequence Analysis

Homologs of *OsGA2ox8* were identified by means of a BLAST search of the NCBI databases (http://www.ncbi.nlm.nih.gov/BLAST/ accessed on 19 March 2019). A multiple sequence alignment was generated with ClustalW and a phylogenetic tree was constructed using the neighbor-joining method with MEGA5 software. The promoter sequences of *OsGA2ox8* (2193 bp, 1301 bp, and 647 bp upstream from the transcription start site) were submitted to the PLACE database (http://www.dna.affrc.go.jp/PLACE/signalscan.html accessed on 16 March 2019) to detect cis-acting elements and predict the regulatory roles of *OsGA2ox8*.

### 5.6. Physiological Analysis

Seedlings of the OE and KO lines and the WT at the three-leaf stage cultured in Yoshida nutrient solution were treated with 20% PEG6000 (~1.1 MPa) for 24 h. The leaves from ten plants were sampled and analyzed for malondialdehyde (MDA) content, soluble sugar content, activities of CAT and SOD, and contents of antioxidants (GSH and AsA). The SOD and CAT activities were estimated as described by Ouyang et al. [66], and MDA content and total soluble sugar concentrations were measured using the methods previously described by Song et al. [67] with minor modifications. The GSH and AsA contents were calculated following the method described by Anderson [68]. All data were analyzed using one-way ANOVA followed by Tukey’s honestly significant difference (HSD) test (*p-*value < 0.05).

### 5.7. Histochemical GUS Staining

The examined tissues of the *OsGA2ox8* *promoter*::*GUS* transgenic lines were collected and incubated in 90% acetone for 1 h. The GUS activity assay was performed in accordance with the method described by Jefferson (1989). The tissues were dehydrated in an ethanol series (70%, 85%, 95%, and 100%) to remove chlorophyll. The stained tissues were viewed with a stereomicroscope (LEICA, Wetzlar, Germany), and the photographs were captured using a digital camera (Canon EOS 600D).

### 5.8. Yeast Two-Hybrid Assay

The coding sequence (CDS) of *OsGA2ox8* was fused to the GAL4 DNA-binding domain (DNA-BD) to generate the pGBKT7-OsGA2ox8 construct. The cDNA library generated from leaves of DK151 [25] at the tillering stage was used for fusion to the GAL4 activation domain by Takara Biotechnology Co., Ltd. (Dalian, China). A yeast two-hybrid assay was performed using the Yeastmaker™ Yeast Transformation System 2 (Clontech, Mountain View, CA, USA) in accordance with the manufacturer’s instructions. Full-length fragments of the candidate interacting proteins were cloned into pGADT7 as prey to confirm the interaction. The pGBKT7-OsGA2ox8 and prey plasmids were co-transformed into yeast strain AH109 and then cultured on SD/–Ade/–His/–Leu/–Trp/X-α-gal plates. A filter assay was performed to test for β-galactosidase activity. The specific primers used to amplify each full-length fragment are listed in Supplementary Table S3.

### 5.9. Subcellular Localization of OsGA2ox8

The CDS of *OsGA2ox8* was fused in frame to the C-terminus of the enhanced GFP CDS under the control of the CaMV 35S promoter. An empty GFP vector was used as the control. The GFP–OsGA2ox8 fusion construct and the empty GFP vector were transformed into rice protoplasts using a polyethylene glycol–calcium-mediated method as described previously [69]. The OsGA2ox8–GFP fusion construct and the empty GFP vector were then infiltrated into *N. benthamiana* leaves using a *Agrobacterium tumefaciens*-mediated transient transformation [70]. The empty GFP vector was used as a control. Fluorescence in the transformed protoplasts and tobacco leaves was captured with a confocal laser scanning microscope (Leica TCS SP5).

### 5.10. Bimolecular Fluorescence Complementation Assay

The full-length *OsGA2ox8* CDS and its candidate interacting proteins were cloned into the vectors pnYFP-X and pcCFP-X, respectively. Bimolecular fluorescence complementation (BiFC) assays were performed following a previously described method [71]. The pair of constructs was co-transformed into the leaves of tobacco by means of *Agrobacterium tumefaciens*-mediated transient transformation using the method described by [70].

### 5.11. Pull-Down Assay

The full-length cDNA of *OsGA2ox8* was cloned into the pCold-TF vector and fused to the N-terminal TF and the C-terminal His-tag. The coding frames of the candidate interacting proteins were cloned into the pGEX6p-1 vector and fused to the N-terminal GST-tag. Purification of the recombinant proteins and the pull-down assays were conducted as described by Qin et al. [72] and Louche et al. [73]. The results were detected by Western blot assays in accordance with standard procedures using the His and GST antibodies, respectively (cell signaling; 1:1000 dilution). The images were visualized with a Tanon-5200 Chemiluminescent Imaging System (Tanon Science and Technology, Shanghai, China).

### 5.12. Transcriptome Analysis

The OE and KO lines and the WT were cultured in Yoshida nutrient solution as previously described herein. Seedlings at the three-leaf stage were treated with 20% PEG6000 (~1.1 MPa) for 24 h. The three uppermost leaves were harvested at one time point for the control and osmotic stress treatment with three replicates for each sample. The transcriptome sequencing and analysis were conducted by Shanghai OE Biotech Co., Ltd. with an Illumina sequencing platform. The FPKM value for each gene was calculated using Cufflinks and the read counts of each gene were obtained using the htseq-count script [74,75,76]. DEGs were identified using the nbinomTest of the DESeq R package and functions estimateSizeFactors with the thresholds *p*-value < 0.05 and |log_2_ Fold Change| > 1 [77]. Kyoto Encyclopedia of Genes and Genomes (KEGG) pathway and gene ontology (GO) enrichment analyses of DEGs were performed with R software based on the hypergeometric distribution [78]. The transcriptome datasets presented in this study can be found below: China National Center for Bioinformation, Genome Sequence Archive (https://bigd.big.ac.cn/gsa/ accessed on 15 March 2021), accession no: CRA004028

### 5.13. Metabolome Analysis

The plant materials used for metabolome analysis were the same as those used for transcriptome analysis with five replicates for each sample. Metabolite extraction and liquid chromatography–tandem mass spectrometry analysis were performed by Shanghai Lu-Ming Biotech Co., Ltd. ACQUITY UHPLC system (Waters Corporation, Milford, MA, USA) and AB SCIEX Triple TOF 5600 System (AB SCIEX, Framingham, MA, USA) were used to analyze the metabolic profile in both ESI positive and ESI negative ion modes. An ACQUITY UPLC BEH C18 column (1.7 μm, 2.1 × 100 mm) was employed in both positive and negative modes.

Progenesis QI data processing software (Waters Corporation, Milford, MA, USA) were used to identify the metabolites based on public databases, such as http://www.lipidmaps.org/ accessed on 1 April 2019, http://www.hmdb.ca/ accessed on 1 April 2019, and custom databases. The differential metabolites were selected with *p*-value < 0.05 and VIP > 1, which were obtained from a two-tailed Student’s *t*-test of the normalized peak areas and the orthogonal partial least squares–discriminant analysis (OPLS–DA) model.

## Figures and Tables

**Table 1:** Analysis of cis-elements in the *OsGA2ox8* promoter sequence.

Cis-Element	Number	Function
DRE2COREZMRAB17	1	AP2/ERF binding site involved in drought stress
DRECRTCOREAT	1	AP2/ERF binding site involved in drought stress
ABRELATERD1	3	cis-acting element involved in the abscisic acid responsiveness
ABRERATCAL	2	cis-acting element involved in the abscisic acid responsiveness
CGTCA-motif	3	cis-acting regulatory element involved in the MeJA-responsiveness
ATCTA	5	AP2/ERF binding site involved in drought stress
GAREAT	1	gibberellin-responsive element
LTRECOREATCOR15	1	cis-acting element involved low-temperature responsiveness
LTRE1HVBLT49	1	cis-acting element involved low-temperature responsiveness
CBFHV	1	cis-acting element involved in the dehydration responsiveness

## Data Availability

The original data presented in the study are included in the article and Supplementary Material. Further inquiries can be directed to the corresponding authors.

## Supplementary Materials

The following are available online at https://www.mdpi.com/article/10.3390/ijms22179107/s1.

## Abbreviations

2-ODDs, oxoglutarate-dependent dioxygenases; AAAAs, aromatic amino acids; AAO, aldehyde oxidase; ABA, abscisic acid; AsA, ascorbic acid; CAT, catalase; CPS, *ent*-copalyl diphosphate synthase; *DDF1*, *DWARF AND DELAYED FLOWERING 1*; DEGs, differentially expressed genes; DT, drought tolerance; FPKM, fragments per kilobase of exon model per million mapped fragments; GA, gibberellin; GFP, green fluorescent protein; GGDP, *trans*-geranylgeranyl diphosphate; GSH, glutathione; H_2_O_2,_ hydrogen peroxide; IAA, indoleacetic acid; JA, jasmonic acid; JAZ, ZIM domain-containing proteins; KO, CRISPR/Cas9 knockout lines; KS, *ent*-kaurene synthase; LOX, lipoxygenase; MDA, malondialdehyde; NCED, 9-*cis*-epoxycarotenoid dioxygenase; OE, overexpression lines; *OsGA2ox8*, gibberellin 2-oxidase 8; PAC, paclobutrazol; PEG6000, polyethylene glycol 6000; SD, standard deviation; SOD, superoxide dismutase; VIP, variable influence on projection; WT, wild type.
