# Supplementary figures and images for: Molecular Dissection of the Gene OsGA2ox8 Conferring Osmotic Stress Tolerance in Rice
Source: Int J Mol Sci. 2021 Aug 24;22(17):9107. doi: 10.3390/ijms22179107 (PMC8430958; doi:10.3390/ijms22179107)

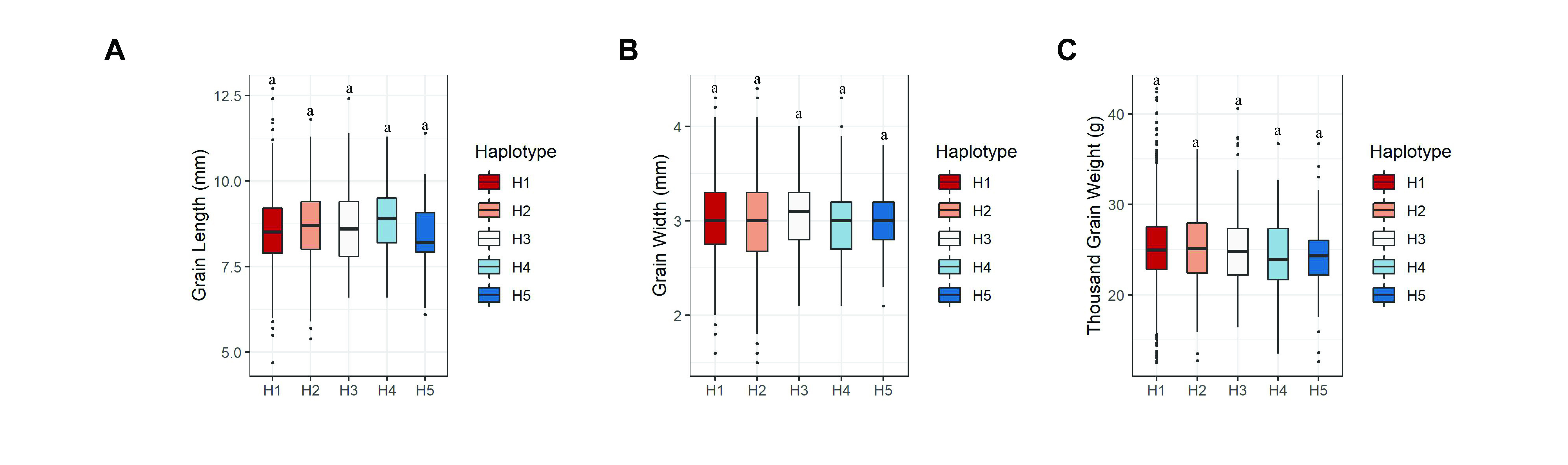

Supplement: Supplementary file 1 [file ijms-22-09107-s001.zip › Supplementary materials/Supplementary Figure 1.jpg]

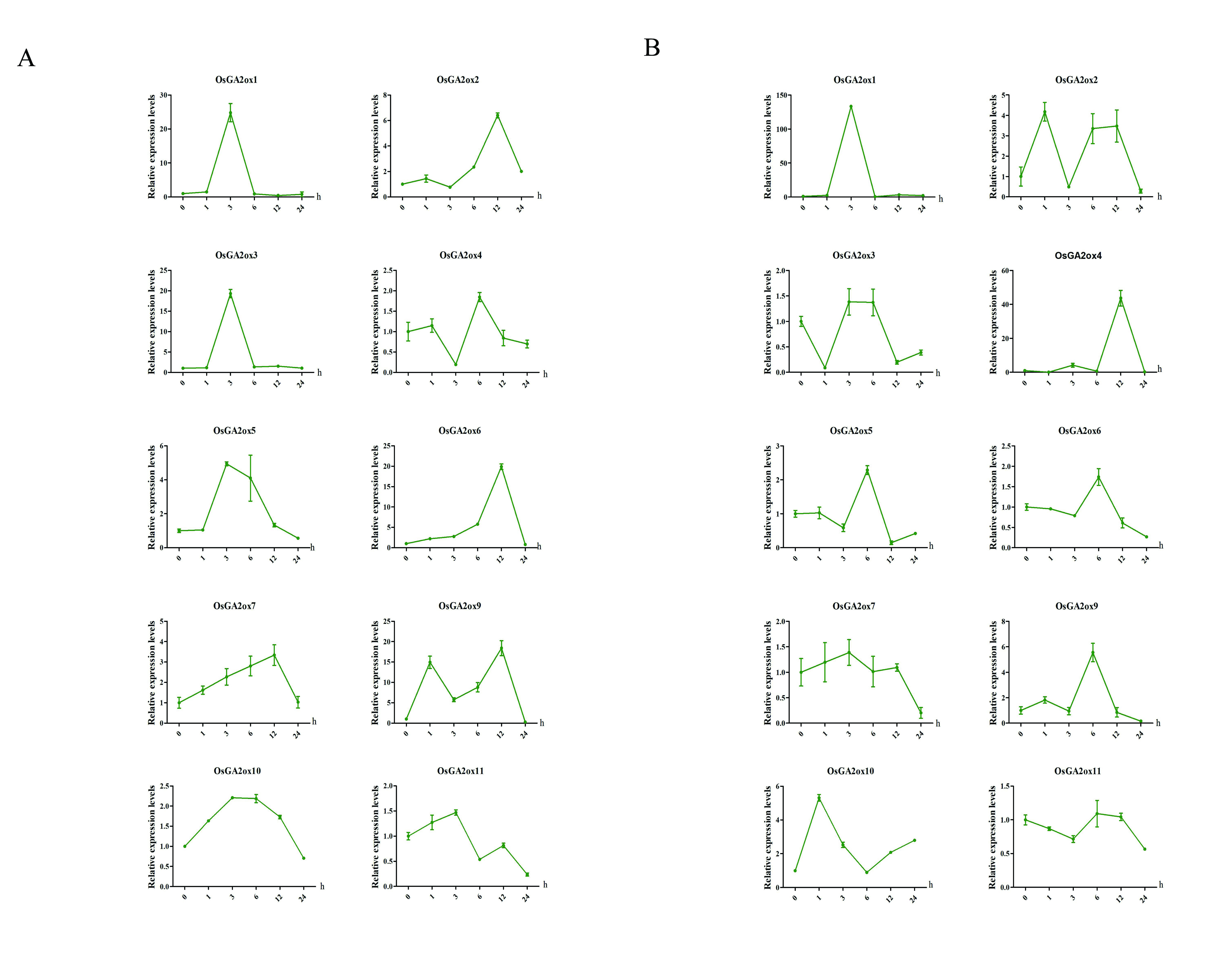

Supplement: Supplementary file 1 [file ijms-22-09107-s001.zip › Supplementary materials/Supplementary Figure 2.jpg]

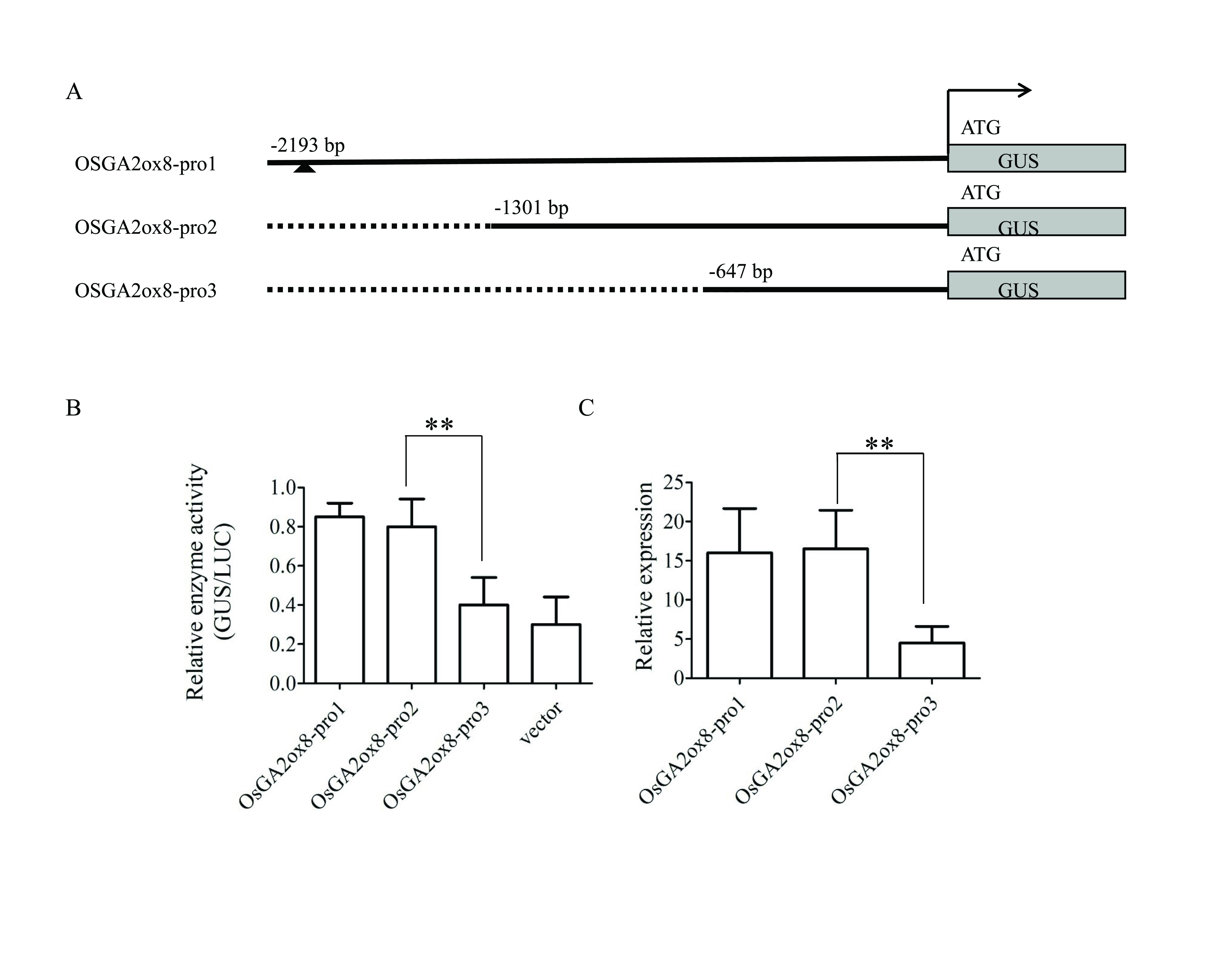

Supplement: Supplementary file 1 [file ijms-22-09107-s001.zip › Supplementary materials/Supplementary Figure 3.jpg]

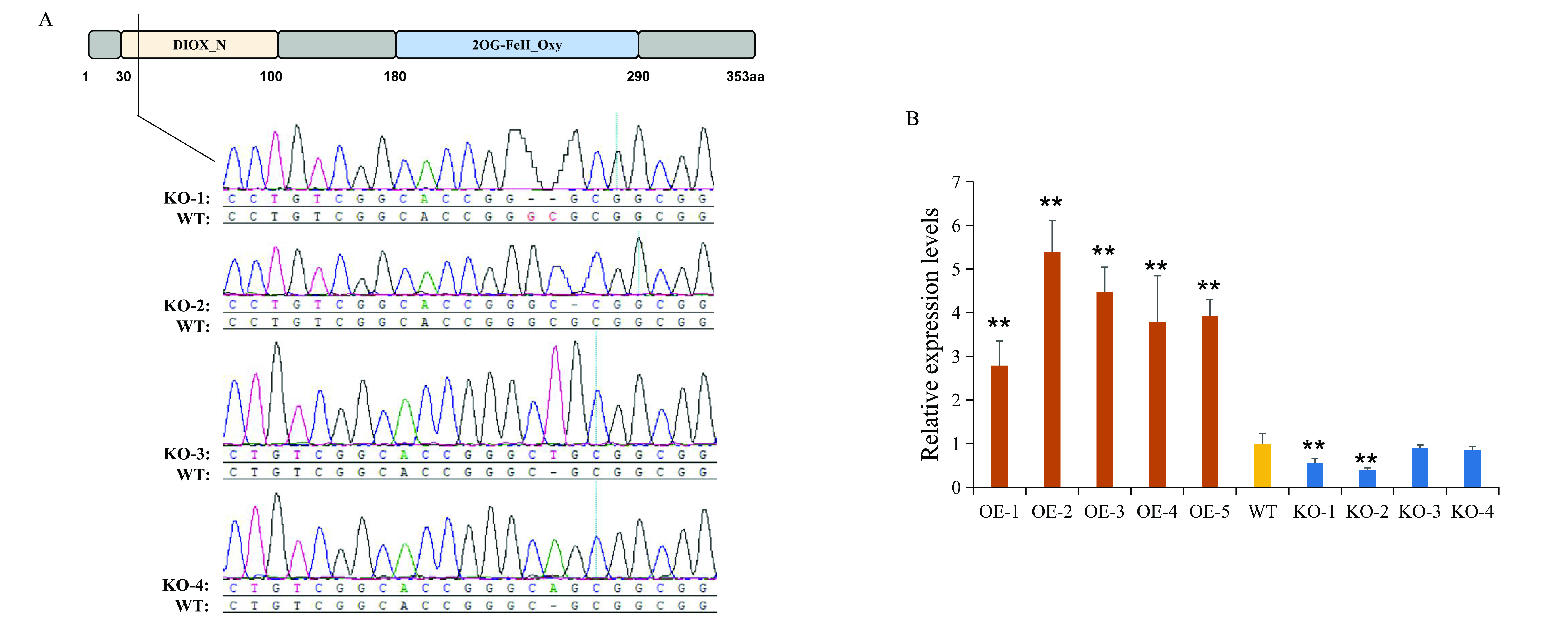

Supplement: Supplementary file 1 [file ijms-22-09107-s001.zip › Supplementary materials/Supplementary Figure 4.jpg]

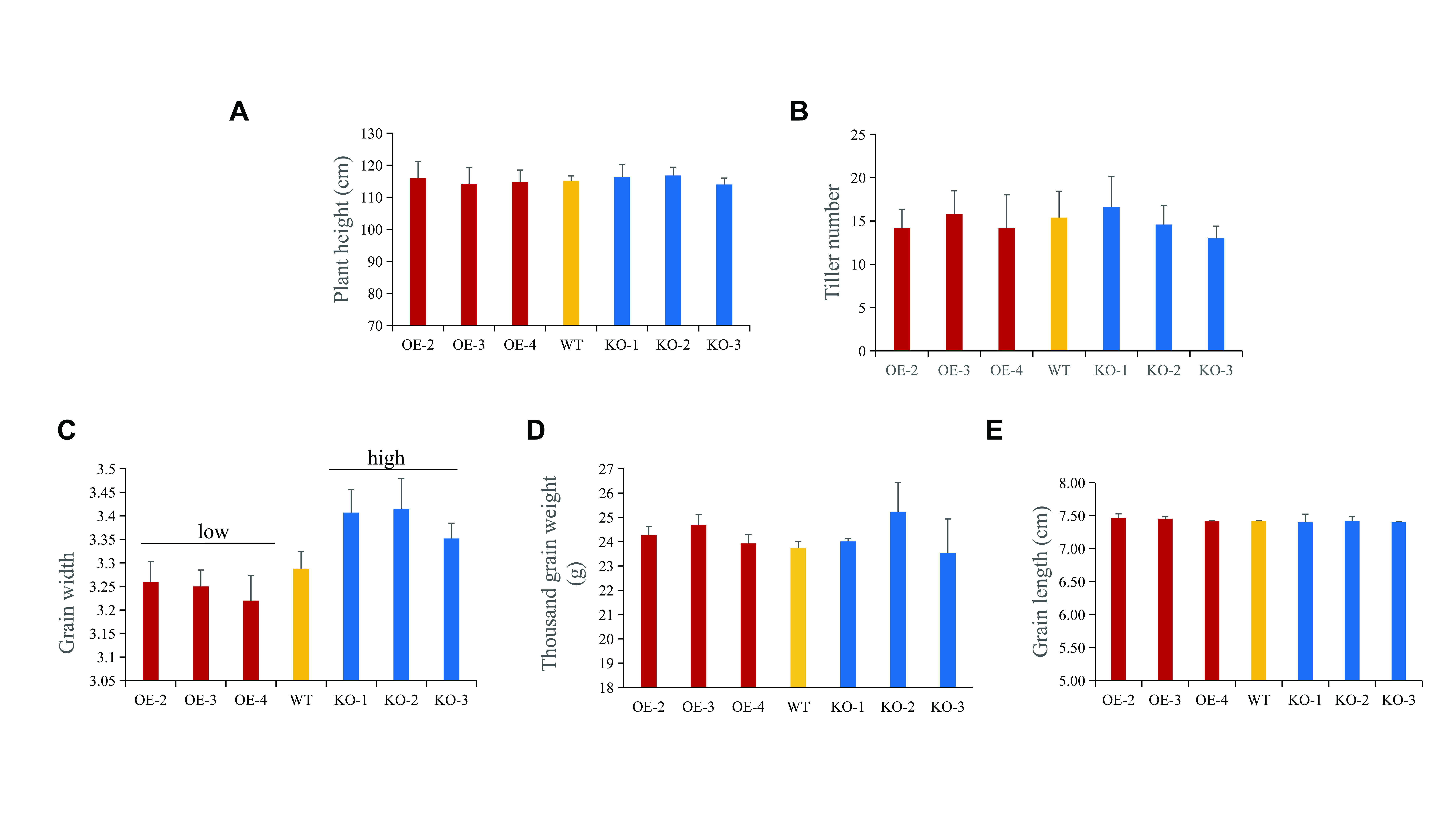

Supplement: Supplementary file 1 [file ijms-22-09107-s001.zip › Supplementary materials/Supplementary Figure 5.jpg]

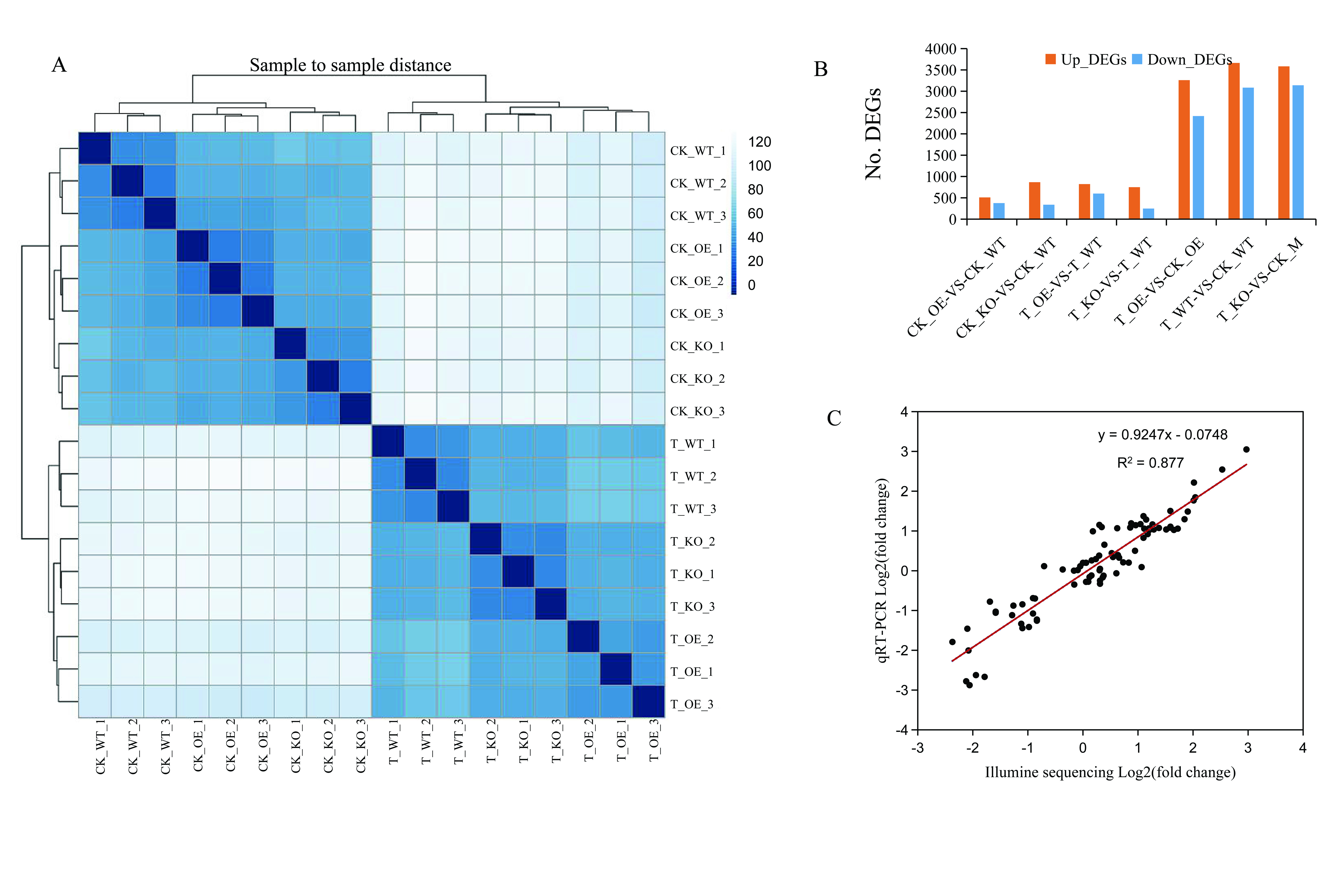

Supplement: Supplementary file 1 [file ijms-22-09107-s001.zip › Supplementary materials/Supplementary Figure 6.jpg]

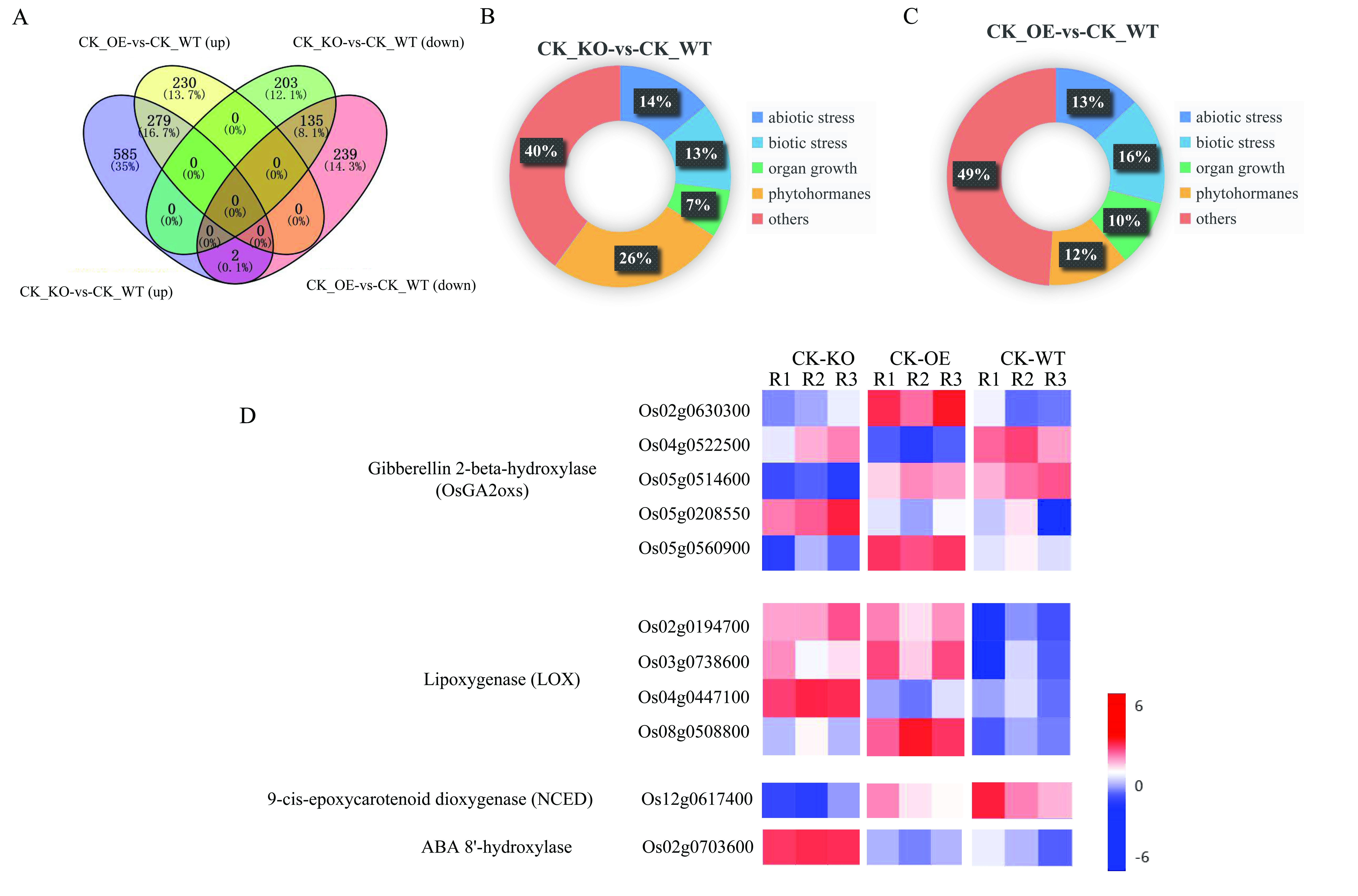

Supplement: Supplementary file 1 [file ijms-22-09107-s001.zip › Supplementary materials/Supplementary Figure 7.jpg]

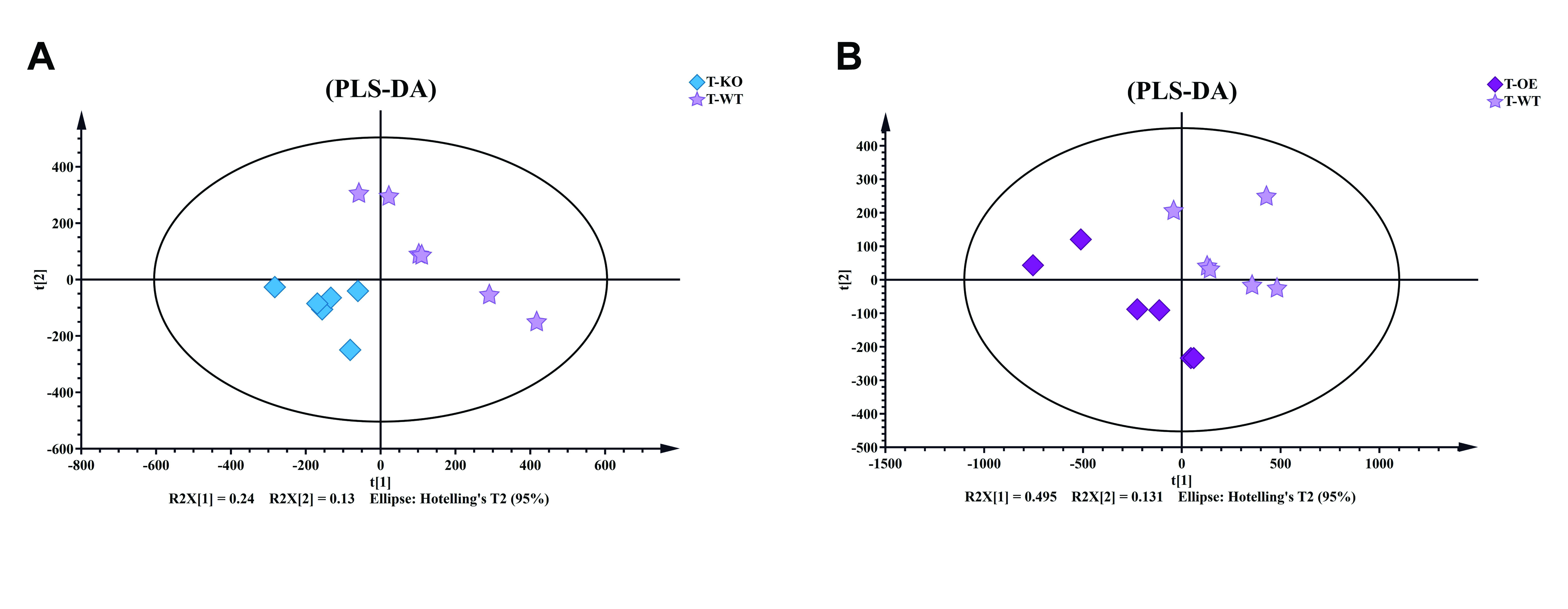

Supplement: Supplementary file 1 [file ijms-22-09107-s001.zip › Supplementary materials/Supplementary Figure 8.jpg]
